# Bioinformatics in Early Cancer Detection

**DOI:** 10.7759/cureus.46931

**Published:** 2023-10-12

**Authors:** Vidya Maheswari Nelakurthi, Priyanka Paul, Amit Reche

**Affiliations:** 1 Public Health Dentistry, Sharad Pawar Dental College and Hospital, Datta Meghe Institute of Higher Education and Research, Wardha, IND

**Keywords:** database, genes, biomarker, cancer, bioinformatics

## Abstract

Bioinformatics is a pretty recent branch of biology that encompasses the use of algebraic, analytic, and computing approaches to the processing and interpretation of biological information. A wide term, "bioinformatics" refers to the use of digital technology to study biological processes using high-dimensional data collected from many resources. The design and testing of the software tools required to evaluate the information are the core of bioinformatics research, which is conducted in great portions in silico and typically involves the synthesis of new learning from available data.

Early diagnosis of cancer results in improved prognosis, but at the same time, it is difficult to conform to diagnosis at a very early stage. The use of DNA microarrays and proteomics studies for large-scale gene expression research has advanced technology, thus elevating the significance of bioinformatics tools. In today's research, wet experimentation and the application of bioinformatics analytics go side by side. Molecular profiling of tumor biopsies is becoming more and more crucial to both cancer research and the treatment of cancer.

## Introduction and background

Introduction

This review mostly provides the researchers with the meaning of bioinformatics and its use in cancer diagnosis and treatment as well as the use of different types of bioinformatic tools and databases. Cancer is one of the major causes of death in all parts of the world and is very difficult to diagnose in its early stages. This review mainly focuses on the use of different types of biomarkers in different types of cancers at the genetic level, which provides a clear idea for a clinical practitioner for better treatment and prognosis. The use of computing and analysis tools in acquiring and interpreting biological data is characterized as bioinformatics [1]. Bioinformatics is a pretty recent branch of biology that encompasses the use of algebraic, analytic, and computing approaches to the processing and interpretation of biological information. The fast increase of internet communication knowledge in the "genomics" and "omics" periods made it overwhelming for laboratory investigators to analyze experimental data during the previous decade. Due to the increase in the need for detailed biological study, complete true biology cannot be satisfied by traditional gene-by-gene research methodologies. Data management and integration across many platforms are required to handle the enormous amounts of information generated by developing sciences such as genome sequencing and microarray chips. Information analysis and reporting are then necessary to gain biotic knowledge for therapeutic results [2]. A wide term, "bioinformatics" refers to the use of digital technology to study biological processes using high-dimensional data collected from many resources. The design and testing of the software tools required to evaluate the information are the core of bioinformatics research, which is conducted in large portions and typically involves the synthesis of new learning from available data [1].

Background

Cancer has been identified as a complex illness that has an impact on many individuals and is considered a common prevalent cause of individual mortality because of the disease's poor identification. Therapy and prognoses might be related to the degree of intensity, time, sites, treatment response and tolerance, cellular proliferation, origination, and pathophysiology knowledge. Cancer bioinformatics is a meaningful and important part of clinical medicine, as well as a key tool and technique in conducting cancer research [3]. The human body produces a lot of genes, proteins, and RNA that are controlled both spatially and temporally to function in a complex web. Because of this problem, the conventional gene-by-gene method is inefficient and unable to offer a comprehensive picture of cellular activity.

Microarray technology has been created to evaluate gene expression from the whole genome in a single experiment [4]. Diagnosis, prognosis, and personalized therapy might all benefit from multi-omics techniques. Adequate bioinformatics tools for organizing, integrating, and interpreting massive and complicated data are required to fulfill the limitations of precision oncology. Due to a tougher policy requirement and the necessity for quick, highly repeatable, and stable processes, we examine the special needs of bioinformatics methodologies and tools that emerge in the scenario of clinical oncology. From the first examination of raw genomic profile data to the automatic preparation of a report, we explain the process of a molecular tumor board and the specialized bioinformatics assistance that it requires [5]. To mediate biological activity, DNA is transcribed into RNA, and RNA is translated into protein, according to the widely accepted fundamental concept of genetics. However, the human genome project shows to our amazement that just 1.5% of the human genome contains protein-coding genes [6-8]. Early genome assemblers were only able to successfully complete the creation of tiny bacterial genomes, but advances in data quality and quantity, together with more sophisticated design methods and processing resources, have made it possible to complete the assembly of more complex eukaryotic genomes [9]. Projects that aim to explain cancer from a global perspective are giving researchers the chance to have greater accessibility to data to integrate and analyze in fresh ways. The ultimate objective of cancer bioinformatics is the development of new treatment and diagnostic methods. Many tools were introduced and developed to know various problems starting from tumor heterogeneity to the analysis of gene mutation [10].

## Review

Bioinformatics in cancer diagnosis

Early diagnosis of cancer causes improved prognosis, but at the same time, it is difficult to confirm the diagnosis at a very early stage due to the lack of top-notch statistical models that take into consideration clinical triage and variations in aggressiveness. Research suggests that identification delay in cancer matters; the evidence for this is mounting, although it is difficult to quantify its effect on survival or fatality [11,12]. The substances present in tobacco are DNA-toxic agents that may have a significant impact on the development and spread of certain cancers [13,14]. Cancer bioinformatics plays a major part in the identification and authentication of biomarkers, specified to early identification, about clinical phenotype, and also measures and observes the prognosis of disease and outcome of treatment and predicts the enhancement of patient's life quality. The incorporation of knowledge on protein annotations, relations, and signaling pathways, and complex biomarkers and novel classes of biomarkers with protein-protein interlinkage were studied. One of the novel approaches is to use effective complex biomarkers to track and assess changes in network biomarkers at various stages and intervals throughout the onset of illnesses. Clinical informatics such as clinical manifestations, patient complaints, treatment histories, biochemical analyses, imaging, pathologies, and other data were anticipated to be connected [15].

The use of DNA microarrays and proteomics studies for large-scale gene expression research has advanced technology, thus elevating the significance of bioinformatics tools. In today's research, wet experimentation and the application of bioinformatics analytics go side by side [16]. Molecular profiling of tumor biopsies is becoming more crucial to both cancer research and the treatment of cancer. Diagnosis, prognosis, and individualized treatment improvements are possible with bioinformatics. Relevant bioinformatics approaches for managing, integrating, and analyzing big, network information are required to fulfill the word precision oncology. Bioinformatics techniques and software that are developed in the context of oncology as a result of the strict monitoring environment and demand for quick, incredibly reproducible, and reliable methods are particularly needed. The molecular tumor board's plan and particular bioinformatics assistance that is needed, from the initial study of fresh molecular profiling information to the computerized making of the report, must be outlined. Numerous clinical studies and genomic tumor boards at specialist cancer centers and medical centers across the globe have employed similar approaches to various extents. Previous initiatives as well as modern ones ought to be examined to integrate tumor boards with certain other top pan-omics patient data, as well as the capability of clinical methodologies to convert molecular discoveries into advice on appropriate treatments. The method used to investigate the genetic basis of cancer is being revolutionized. Instead of concentrating on specific genes, researchers are now investigating important areas of the expressed genome. The quantity of data saved in the patient record and the amount of molecular data produced from the testing facility are growing at an incredible rate. To obtain a fresh understanding of the genetics of cancer, it is essential to find innovative approaches for combining these data. As a result, bioinformatics, the fusion of biology, information science, and computation, continues to become an essential part of cancer research [17].

Bioinformatics databases and tools

It is very important to collect useful and important data related to the study before using any technique. This process of collection of data is called data mining [18]. Oncomine is the name of the software system utilized in the data mining procedure. All free-of-charge cancer microarray data is meticulously curated, examined, and made accessible through the database and information mining technology known as Oncomine [19]. The tools of bioinformatics include the Database for Annotation, Visualization and Integrated Discovery (DAVID), Gene Ontology (GO), Surveillance, Epidemiology, Results Program (SEER), and Gene Expression Profiling Iterative Analysis (GEPIA) [20].

Classification of databases

Databases are classified as follows: databases harboring gene/microRNA expression profiles, databases for copy number variations (CNVs), DNA mutation detection databases, epigenetic profiles databases, databases with integrative analyses, and databases with other data types [21]. There are four important biological data: DNA, RNA, protein sequences, and microarray images. The first three of them are text data, and the fourth one is a digital image [22].

MicroRNA Expression Profiles

MicroRNAs are a newly identified type of small (22 nucleotides) naturally existing molecules of RNA that suppress after-transcriptional control of gene expression. As such molecules have been discovered to play vital functions in several different biological functions and to be aberrantly displayed in many forms of cancer, including blood cancer, there has been an increase of interest in the subject of microRNA. Tumor suppressor molecules such as miR-15a and miR-16-1 and oncogenic molecules such as the miR-155 and miR-17-92 cluster can both be found in cancer-associated microRNAs. Lawrie highlighted the fast-mounting evidence for the crucial involvement microRNAs play in both blood malignancy and hematopoiesis, with a special emphasis on lymphoma [23]*.*

Databases for Copy Number Variations (CNVs)

A specific type of chromosomal structural reorganization known as copy number variation (CNV) has been primarily identified in this decade using scientific technologies. Experts have discovered that CNVs are common across a wide range of animals, and mounting data suggests a strong connection between CNVs and complicated illnesses. The study of genomic structural changes has started to offer certain crucial hints about the pathologic origins of illnesses and the course of the condition. Experimental data, however, have not yet been properly gathered or organized, and the majority of the published investigations have concentrated on a specific illness [24].

Discussion

Bioinformatics in Lung Cancer

Lung cancer is the common cause of cancer mortality; however, there are still no trustworthy molecular indicators. To find biomarker genes for non-small cell lung cancer, which has a poor prognosis and a high risk of recurrence, Kim et al. employed several clinical samples, combining bioinformatics analysis of the public gene expression profiles with clinical validation. They selected 20 genes for field measurements by semiquantitative RT-PCR after meta-analyzing the serial analysis of gene expression (SAGE) and expressed sequence tags (EST) data. After that, two extremely likely new biomarkers (*CBLC* and *CYP24A1*) were discovered by applying quantitative RT-PCR to seven genes that had been identified as prospective diagnostic markers (*CBLC*, *CYP24a1*, *ADLH3a1*, *AKR1B10*, *S100P*, *PLUNC*, and *LOC147166*) [25]. Maharjan et al., using the R software utility GEO2R, the differentially expressed genes (DEGs) for lung cancer were found in the Gene Expression Omnibus (GEO) database [26]. A total of 547 DEGs (133 upregulated and 414 downregulated) were extracted from treatment trials, while 407 DEGs (255 upregulated and 153 downregulated) were obtained from non-treatment studies. They used functional networks created using DEGs to produce two Cytoscape programs, CytoHubba and MCODE, to find biomarker genes. This study identified two unique sets of biomarker genes, each set having 16 genes, one from non-treatment trials and the other from treatment studies. The results of the survival study demonstrate the prediction potential of the majority of non-treatment biomarker genes by demonstrating that low-expression groups had a higher chance of surviving than high-expression groups, while the majority of therapy indicators can predict outcomes by suggesting that high expression group has a greater survival rate than low expression group [26].

Bioinformatics in Breast Cancer

Using serial analysis of gene expression (SAGE), cell-type-specific cell surface indicators, and magnetic resonance imaging, Allinen et al. characterized the full transcriptome of every cell type making up healthy breast tissue as well as in situ and metastatic breast carcinomas. Beads were utilized for the quick isolation of each step. Their findings imply that all cell groups undergo modifications, but only cancer epithelial cells have genetic changes identified [27].

Bioinformatics in Liver Cancer

After lung cancer, liver cancer accounts for the majority of cancer-related fatalities. Using a mouse hepatoblast model and RNAi, Sawey et al. carried out a forward genetic screening under the direction of human hepatocellular cancer amplification data. They discovered that the selected susceptibility to *FGF19* inhibition was caused by overexpression. Since *CCND1* and *FGF19* are both equally significant driver genes of the 11q13.3 amplicon in liver cancer, 11q13.3 amplification may serve as a useful biomarker for individuals who are expected to respond favorably to anti-FGF19 treatment [28].

Bioinformatics in Brain Tumors

By employing this categorization to separate medulloblastomas from other histologically comparable brain tumors, Pomeroy et al. were able to anticipate the treatment activity of medulloblastomas [29]. Moreover, the molecular profile demonstrated that medulloblastomas and primitive neuroectodermal tumors (PNETS), two forms of brain cancers frequently regarded as single entities, are physiologically separate from one another. The medulloblastoma gene expression pattern revealed unanticipated participation of the sonic hedgehog signaling pathway and suggested cerebellar granule cells as their cell of origin. Bredel et al. also applied genomic network knowledge to the investigation of critical activities and addressing glioma genesis to employ gene transcription profiling in the biology view of human gliomas [30]. Several cancer investigators have used microarray technology to study multiple myeloma. The morphological uniformity of multiple myeloma was verified by Claudio et al. [31]. Locati et al. used self-organizing map techniques to organize publicly accessible HPV+ cancer information and derived gene signatures related to three different subgroups of the illness as a substitute strategy and to truly comprehend how gene groups may connect with prognosis [32]. A 10-gene proposed methodology of relapse duration and prognosis in epithelial ovarian cancer was discovered by Lu et al. using a support vector machine learning algorithm to examine the information from the cancer cell line encyclopedia. This model was verified on two different pieces of information [33].

Bioinformatics in Oral Cancer Detection

Oral cancer is one of the most commonly seen neoplasms in the head and neck region and has a poor prognosis, and among oral cancer, oral squamous cell carcinoma (OSCC) is the most common [34,35]. Kumar et al. compared the top-ranked genes with the genes corresponding to strongly enriched GO keywords relevant to oral cancer. A total of 39 prospective oral cancer target genes were identified. Initial analysis of research and experimental data revealed 29 genes to be associated with OSCC. They proposed a function for the chosen candidate genes in the invasion and metastasis in OSCC following a thorough pathway analysis. Using immunohistochemistry (IHC), they further verified their hypotheses and discovered that in the OSCC specimens, *FLNA* was elevated, whereas *ARRB1* and *HTT* were downregulated [35]. Nakashima et al. found that the miR-1290 expression level in the plasma of oral squamous cell carcinoma patients is lower than in healthy individuals. However, circulating miR-1290 status has been proposed as a promising biomarker for evaluating both overall survival and clinical response to chemoradiotherapy in patients with oral squamous cell carcinoma [36]. Differentially expressed genes, hub proteins, and pathways demonstrated a strong correlation with OSCC development. Thorough investigation using bioinformatics is necessary for understanding the underlying process of OSCC advancement. Important genes and pathways may serve as OSCC therapy management goals [37].

Bioinformatics in Ovarian Cancer

Ovarian cancer is the most prevalent and main cause of female death globally among numerous gynecological cancers. The pathophysiology and underlying causes of illness development remain unexplained despite substantial investigation. Different non-coding RNAs have been recognized as key regulators in the development of ovarian cancer. Beg et al. highlighted the significance of several ncRNAs, which have a strong promise as a therapeutic strategy for the treatment of ovarian cancer [38].

## Conclusions

This selection of articles highlights a variety of methods and data points that demonstrate the breadth of bioinformatics methods being established to solve challenging issues such as how to more accurately predict treatment benefits and how to prioritize lead compounds with the potential to impact the tumor immune microclimate. The articles show how machine learning techniques may be applied to significantly enhance cancer detection. Indeed, we might ultimately aspire to enhance research efficiency and make significant changes to patient health as we get a better knowledge of how various machine learning techniques are best suited to explore the important problems described in the article.
